# Psychiatric Disorders and Their Impact on Herpes Zoster Incidence: Five Years of Data from Turkiye

**DOI:** 10.3390/jcm13237401

**Published:** 2024-12-04

**Authors:** Caner Yeşiloğlu, Cihan Yeşiloğlu, Lut Tamam, Mehmet Emin Demirkol

**Affiliations:** 1 Department of Psychiatry, Faculty of Medicine, Çukurova University, Balcalı Campus, 01330 Adana, Türkiye; ltamam@gmail.com (L.T.); emindemirkol@gmail.com (M.E.D.); 2 GSK, Medical Department, 34330 Istanbul, Türkiye; cihan.yesiloglu@istanbul.edu.tr

**Keywords:** herpes zoster, incidence, psychiatric disorders, shingles

## Abstract

**Background/Objectives**: This study investigates the relationship between psychiatric disorders and herpes zoster (HZ). Emergency and outpatient admissions to Kırşehir Education and Research Hospital between 2019 and 2023 were examined. Psychiatric disorders are known in the literature as one of the factors that weaken the immune system, thereby increasing the risk of infection. **Methods**: In our study, the past diagnoses and sociodemographic data of all individuals over the age of 18 who applied to the Kırşehir Education and Research Hospital between 2019 and 2023 were obtained from hospital system records. Patients receiving immunosuppressive treatment or those with diseases that could affect immune system function were excluded from the study. The sample was divided into two groups: those with and without psychiatric disorders diagnosed with HZ. Sociodemographic features, change of HZ frequency over the years, and differences among psychiatric diagnosis subgroups were examined between the groups. **Results**: Individuals with psychiatric diseases had a significantly higher frequency of HZ compared to those without psychiatric diseases (0.0040% vs. 0.0016%, *p* < 0.001). No significant difference was found in the frequency of HZ among different types of psychiatric disorders (*p* = 0.60). Additionally, no statistically significant difference was found in the median age between the groups (*p* = 0.11). In terms of gender distribution, it was determined that women were more frequently diagnosed with HZ compared to men. **Conclusions**: The frequency of HZ was found to be higher in individuals with psychiatric disorders. However, further research is needed to explore the association between specific psychiatric diagnoses and HZ frequency.

## 1. Introduction

Varicella zoster virus (VZV) is an alpha herpesvirus with a double-stranded DNA genome. VZV infects humans without an animal reservoir, targeting T lymphocytes, epithelial cells, and ganglia. The primary infection causes chickenpox, and after this infection, VZV remains latent in the ganglionic neurons [1].

VZV primary infection often leads to a clinical presentation that the immune system can control. Reactivation of VZV replication and its spread to the skin, innervated by these neurons, result in the clinical presentation of herpes zoster (HZ). HZ can be complicated by chronic pain, neurological and ocular disorders (meningoencephalitis, myelitis, cranial nerve palsies, vasculopathy, keratitis, and retinopathy), and multi-visceral and gastrointestinal disorders such as ulcers, hepatitis, and pancreatitis [2,3]. Older age and weakened immune function are risk factors for VZV infection complications [3]. Notably, HZ may also be present in the absence of the characteristic cutaneous lesions, a condition termed “zoster sine herpete”. This atypical presentation has been associated with greater pain severity and an increased need for analgesic use [4].

Although the mechanism involved in the reactivation of latent VZV is not fully understood, there is evidence that decreased cellular immunity against VZV predisposes to HZ formation [5,6]. Additionally, suppressed immune responses have often been associated with psychiatric disorders (mental disorders). Animal models have shown that cellular and humoral immune systems are impaired in highly anxious mice, leading to a weakened immune response [7]. Research has also suggested that psychological stress, anxiety, and depression negatively affect immune function and increase susceptibility to infection [8,9,10,11]. Irwin et al. stated that major depression is particularly associated with a marked decline in VZV-specific cellular immunity [12]. Although this evidence suggests impaired cellular immunity in individuals with psychiatric disorders, data on the risk of HZ in those with psychiatric diseases are limited [1].

There is substantial evidence in the literature of an important relationship between psychiatric disorders and immune system function. It is well established that psychiatric conditions such as chronic stress, depression, anxiety, and psychotic disorders suppress immune response, making individuals more susceptible to viral infections [13]. The reactivation of latent viruses such as HZ has been found to be associated with psychological stress and a decline in immune function [14]. Increased HZ risk in psychiatric disorders may be influenced by both direct immune suppression and self-care deficits associated with these conditions. Individuals with severe psychiatric disorders, such as schizophrenia and bipolar disorder, are reported to experience challenges in accessing healthcare services and maintaining health monitoring, which makes them more vulnerable to infections [15]. The number of studies on the association between psychiatric disorders and HZ is quite limited. Two studies have examined the impact of psychological stress on HZ risk. In a retrospective case-control study, 101 patients with HZ were reported to have experienced more frequent negative events, identified as stressors, two to three and six months before the onset of the skin rash compared to controls of the same age group [10]. Another prospective cohort study involving 2568 individuals over the age of 65 showed that negative life events were weakly associated with an increased risk of subsequent HZ, with a reported relative risk of 1.38 [16]. However, since these studies did not include specific clinical diagnoses of psychiatric disorders, their findings cannot be directly extrapolated to the association between psychiatric disorders and the emergence of HZ [1].

This study aims to determine the annual prevalence of HZ in Turkish patients and among those with and without psychiatric disorders to demonstrate the susceptibility to HZ in individuals with psychiatric disorders. Considering the variability in annual prevalence across populations, it is believed that annual prevalence studies conducted in different populations will contribute to the literature [17].

## 2. Materials and Methods

### 2.1. Study Design

This study was conducted among cases admitted to Kırşehir Education and Research Hospital, a major healthcare facility in the Central Anatolia Region, providing services to both a large metropolitan area and neighboring provinces. The hospital receives approximately 1,100,000 outpatient visits per year, with about 40,000 annual visits from 10,000 individuals to its psychiatry department. The sample of our study consists of 1,409,739 individuals aged 18 and over who visited the hospital between 2019 and 2024 (a total of 3,814,106 visits from individuals aged 18 and over). The age and gender distributions of the group were analyzed, and participants were divided into subgroups based on median age and gender. To calculate the annual prevalence of HZ for each year from 2019 to 2024, we divided the number of newly diagnosed HZ cases by the total number of unique hospital visitors for that year. This rate was expressed as cases per 1000 persons, allowing for a year-by-year comparison of HZ trends across the study period. This retrospective design allowed for the comprehensive analysis of a large dataset over a five-year period.

### 2.2. Sample Selection

The inclusion criteria focused on individuals over 18 years of age who did not have any of the excluded comorbidities. The exclusion criteria were selected to ensure a more homogenous sample without conditions that could interfere with immune function or HZ susceptibility. Patients with an immunodeficiency diagnosis or cancer were excluded from the study.

### 2.3. Data Collection

The sociodemographic data and diagnoses of the participants, determined according to ICD-10 codes, were retrospectively retrieved from the hospital system. The diagnosis of HZ was made clinically and/or through appropriate laboratory analysis. The ICD-10 codes used for diagnoses included: B02: herpes zoster, B02.0: herpes zoster with meningitis, B02.1: herpes zoster with other nervous system complications, B02.2: herpes zoster with ocular complications, B02.3: herpes zoster with disseminated disease, B02.7: disseminated zoster, B02.8: zoster with other complications, B02.9: herpes zoster without complications; F32: depressive episode, F32.0: major depressive disorder, single episode, mild, F32.1: major depressive disorder, single episode, moderate, F32.2: major depressive disorder, single episode, severe without psychotic features, F32.3: major depressive disorder, single episode, severe with psychotic features, F32.4: major depressive disorder, single episode, in partial remission, F32.5: major depressive disorder, single episode, in full remission, F32.9: depressive disorder, unspecified; F41: anxiety disorders, F41.0: panic disorder [episodic paroxysmal anxiety], F41.1: generalized anxiety disorder, F41.2: mixed anxiety and depressive disorder, F41.3: other mixed anxiety disorders, F41.8: other specified anxiety disorders, F41.9: anxiety disorder, unspecified; F20: schizophrenia, F20.0: paranoid schizophrenia, F20.1: hebephrenic schizophrenia, F20.2: catatonic schizophrenia, F20.3: undifferentiated schizophrenia, F20.4: post-schizophrenic depression, F20.5: residual schizophrenia, F20.6: simple schizophrenia, F20.8: other schizophrenia; F29: unspecified psychosis not due to a substance or known physiological condition; F31: bipolar affective disorder, F31.0: bipolar disorder, current episode hypomanic, F31.1: bipolar disorder, current episode manic without psychotic features, F31.2: bipolar disorder, current episode manic with psychotic features, F31.3: bipolar disorder, current episode mild or moderate depression, F31.4: bipolar disorder, current episode severe depression without psychotic features, F31.5: bipolar disorder, current episode severe depression with psychotic features, F31.6: bipolar disorder, current episode mixed, F31.7: bipolar disorder, currently in remission, F31.8: other bipolar disorder, F31.9: bipolar disorder, unspecified. ICD-10 codes were used to ensure standardization and accuracy in diagnosis reporting across the sample.

The hospital admission times and recurrent visits of the cases were recorded. The gender, age, and diagnosis frequencies of individuals diagnosed with HZ and those diagnosed with both HZ and psychiatric disorders were compared. In cases where individuals were diagnosed with HZ more than once, duplicate records were excluded from the analyses.

### 2.4. Statistical Analysis

The data were analyzed using IBM SPSS version 25.0 (IBM Corp., New York, NY, USA). Descriptive statistics for categorical variables were summarized as frequencies and percentages. The normality of the distribution of the groups was checked using skewness and kurtosis values. A group was considered to have a normal distribution if these values fell between −1.5 and +1.5 [18]. In cases where continuous variables showed a normal distribution, mean values were used. To compare categorical variables between the groups, the Chi-square test was applied when each cell in the contingency table had values greater than five, and Fisher’s exact test was used when the values were less than five. To compare the means of two independent groups, an independent *t*-test was applied when the data showed a normal distribution. When the data did not show a normal distribution the Mann–Whitney U test was applied for comparisons.

A linear regression model was constructed to define the trend in annual case counts. In this model, the dependent variable (HZ case count) and the independent variable (year) are analyzed to determine the relationship, as represented by the slope and correlation coefficient (R). The correlation coefficient indicates the direction and strength of the trend, while the slope reflects the amount of annual change. A logistic regression model was also employed to assess the impact of gender and psychiatric diagnoses on the likelihood of developing shingles, with results reported as odds ratios, Wald values, and 95% confidence intervals to determine statistical significance.

### 2.5. Ethics

Ethics committee approval for the study was obtained from the hospital administration and Kırşehir Provincial Health Directorate, followed by approval from the Non-Interventional Research Ethics Committee of Çukurova University.

## 3. Results

### Figures, Tables, and Schemes

Table 1 presents the annual number of unique hospital visitors and individuals diagnosed with HZ between 2019 and 2023, with a total of 2476 unique cases diagnosed over the five-year period. The annual prevalence of HZ was calculated based on unique individuals visiting the hospital each year, excluding repeat visits by the same person. Thus, the values in Table 1 reflect the incidence per 1000 unique hospital visitors, ensuring that each data point represents individual cases rather than visit frequency. While the population showed a similar gender distribution, the rate of HZ diagnosis was higher in women than in men. The female-to-male ratio among individuals diagnosed with HZ varied across years. Overall prevalence rates did not show a significant change annually, with the highest prevalence occurring in 2021. These data reflect the distribution of HZ diagnoses over the years and gender differences.

**Figure 1:**
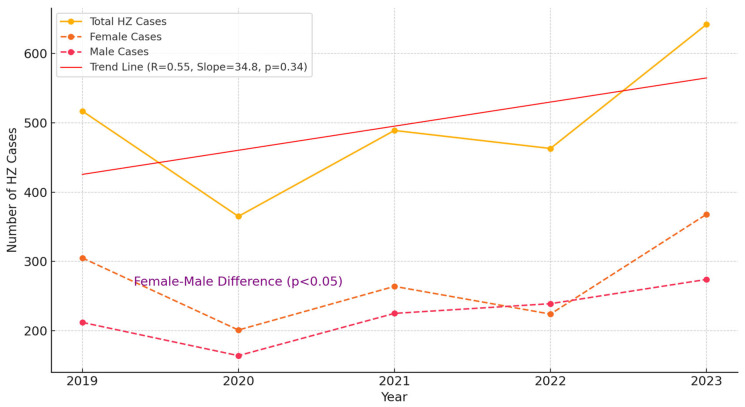
Trend of total herpes zoster cases over the years.

Figure 1 presents the trend of HZ diagnoses over the years and the differences between genders. Trend analysis revealed no statistically significant difference in the number of individuals diagnosed with HZ over the years (R = 0.55, slope = 34.8, *p* = 0.34). When examining gender differences, it was shown that the frequency of HZ diagnoses was statistically significantly higher in women than in men (*p* < 0.05). Table 2 compares the average age, gender ratios, and psychiatric diagnosis frequency of individuals with and without psychiatric diagnoses alongside HZ. The average age of the group with psychiatric disorders was 51.6, while that of the group without psychiatric disorders was 56.3. No statistically significant difference was found between the groups in terms of age (*p* = 0.27). However, there was a statistically significant difference in the frequency of HZ diagnoses between individuals with psychiatric diagnoses (0.0040) and those without psychiatric diagnoses (0.0016) (χ^2^ = 3678.08, *p* < 0.001). This finding indicates that the frequency of HZ diagnoses is significantly higher in individuals with psychiatric disorders than in those without.

Table 2 compares the average age, gender ratios, and psychiatric diagnosis frequency of individuals with and without psychiatric diagnoses alongside HZ. The median age of the group with psychiatric disorders was 50, while that of the group without psychiatric disorders was 55. The median age for all individuals diagnosed with HZ was also 55. No statistically significant difference was found between the groups in terms of age (*p* = 0.11). However, there was a statistically significant difference in the frequency of HZ diagnoses between individuals with psychiatric diagnoses (0.0040) and those without psychiatric diagnoses (0.0016) (χ^2^ = 3678.08, *p* < 0.001). This finding indicates that the frequency of HZ diagnoses is significantly higher in individuals with psychiatric disorders than in those without.

**Figure 2:**
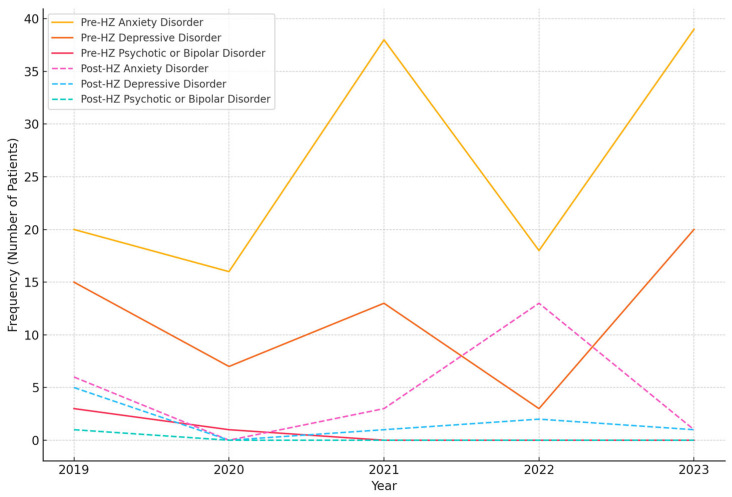
Frequency of psychiatric diagnosis groups accompanying herpes zoster.

Figure 2 presents the frequency of psychiatric diagnosis groups accompanying HZ and the proportion of individuals diagnosed with psychiatric disorders after being diagnosed with HZ. No statistically significant difference was found among the diagnostic groups.

Table 3 examines the distribution of anxiety disorders, depressive disorders, psychotic disorders, and bipolar disorder in individuals diagnosed with both HZ and psychiatric disorders. The frequency of HZ diagnoses was higher in each psychiatric diagnosis group compared to the healthy population (*p* < 0.001).

Table 4 presents the logistic regression analysis results for shingles risk factors. The presence of anxiety, depression, and psychosis or bipolar disorder was associated with a higher risk of shingles. Specifically, anxiety was associated with a higher risk of shingles (odds ratio = 3.23), depression with a moderate increase in risk (odds ratio = 2.1), and psychosis or bipolar disorder with the highest increase (odds ratio = 6.55); all of these effects were statistically significant. Additionally, female gender was associated with an increased risk of shingles (odds ratio = 1.39), which was also statistically significant. Furthermore, being in the age group of 55 years and older was associated with a 2.55-fold increase in the risk of shingles, and this effect was also statistically significant.

## 4. Discussion

HZ is a latent viral infection that becomes more likely to manifest in individuals with weakened immune systems, in the context of infectious diseases, aging, immunosuppressive treatments, chronic diseases, and psychological stress. The incidence of HZ in Europe has been reported to be 2–4 per 1000 people under the age of 50, 7–8 per 1000 people over the age of 50, and up to 10 per 1000 people over the age of 80. The Centers for Disease Control and Prevention (CDC) in the United States estimates that one in three individuals will develop HZ at least once in their lifetime [19]. In single-center studies conducted in Turkey, the rate of HZ in dermatology patients has been reported to range between 0.43% and 1.56% [20]. VZV reactivation is thought to be closely linked to impaired cellular immunity, which plays a crucial role in maintaining viral latency. In psychiatric disorders, factors such as chronic stress, anxiety, and depression are known to affect immune function, potentially leading to immune dysregulation and diminished VZV-specific cellular immunity. This includes impaired activity of Th17 cells, which are involved in pro-inflammatory responses, and regulatory T cells (Treg), which help maintain immune homeostasis and prevent excessive inflammatory responses. Disruptions in these immune cell functions may contribute to an increased risk of VZV reactivation in individuals with psychiatric illnesses. Future research focusing on these immune pathways could clarify the underlying mechanisms linking psychiatric conditions to increased susceptibility to VZV reactivation [12].

In our study, the annual prevalence of HZ over five years was found to range between 0.16% and 0.20% (with an average of 0.19%). Since our study excluded immunosuppressed individuals and those with certain chronic diseases, this rate is considered consistent with the literature [21]. The highest incidence was recorded in 2021, which, as noted in the literature, may be associated with the increased frequency of HZ following COVID-19 [19]. In 2020, Turkey implemented a series of mandatory lockdowns and restrictions due to the COVID-19 pandemic, which led to a substantial decrease in non-emergency hospital visits nationwide. During these lockdown periods, individuals were advised to avoid hospital admissions unless they required urgent care, resulting in a noticeable reduction in hospital admission rates. Consequently, the decrease in herpes zoster (HZ) cases observed in 2020 aligns with the reduced number of hospital visits that year, rather than an actual decline in HZ prevalence among the general population. This factor is crucial for understanding the trends in Table 1 and Figure 1, reflecting the impact of pandemic-related restrictions. [22,23,24]. It has been reported that the presence of psychiatric disorders may impair immune response, making it a risk factor for HZ. However, studies on this subject are limited. The most important finding of our study is that the incidence of HZ was significantly higher in individuals with psychiatric disorders compared to those without (*p* < 0.001). Our results provide epidemiological evidence that supports previous findings regarding immunological changes in psychiatric disorders [25]. Psychological conditions such as anxiety and stress impair the immune response by reducing T lymphocyte counts and immunoglobulin concentrations. Psychosocial stress reduces immunological control over VZV, and psychological stress has been identified as a risk factor for HZ (RR = 1.47) [26]. In our study, the incidence of HZ among individuals with psychiatric diagnoses at the Kırşehir Education and Research Hospital between 2019 and 2023 was found to be 0.030–0.053%, which was significantly higher than in those without psychiatric disorders. The impaired immune response in psychiatric disorders may explain the increased susceptibility to latent viral infections, including HZ.

Yang et al. followed patients aged 60 and older for two years and found that the risk of HZ was 1.34 times higher (*p* = 0.026) in those diagnosed with psychosis, 1.42 times higher in those with personality disorders, and 1.53 times higher in those with depressive or anxiety disorders, as well as other psychiatric illnesses [1]. In our study, anxiety disorder was the most common psychiatric disorder accompanying HZ, but this can be explained by the fact that 72% of participants had anxiety disorder. The relationship between HZ frequency, gender, age and psychiatric disorders was demonstrated. The strongest effect on HZ frequency was observed in patients diagnosed with psychotic and bipolar disorders, followed by anxiety disorders, depressive disorders, older age, and female sex. This may be explained by reduced immunity and impaired self-care in psychotic and bipolar disorders [27]. An alternative explanation is that antipsychotic medications used in this group may suppress cellular immune functions, leading to viral reactivation. This immunosuppressive effect of antipsychotics may increase the risk of HZ [15].

The literature reports that over 95% of individuals aged 50 and above are seropositive for VZV, meaning they are at risk of developing HZ [28]. Age is one of the most important identified risk factors for HZ. The lifetime risk of developing HZ is between 25% and 30%, and this risk increases to 50% in individuals over the age of 80. It has been particularly noted that the frequency of HZ increases after the age of 50, with the average age of HZ onset in adults being 59.4 years, and 68% of cases occurring in individuals aged 50 and above [29]. Given the significant burden of HZ in older populations, highly efficient HZ vaccines provide a crucial preventive strategy, particularly for older adults and vulnerable individuals, such as those with psychiatric disorders [30]. To the best of our knowledge, no study has been conducted in the Turkish population regarding the age at which HZ is diagnosed in individuals with psychiatric disorders. In our study, the median age of individuals with psychiatric disorders was 50, while it was 56 in those without psychiatric disorders. The difference in median age was not statistically significant. The earlier age of HZ onset in individuals with psychiatric disorders may be explained by the impairment of immune response caused by psychiatric disorders. However, the small number of individuals diagnosed with both HZ and psychiatric disorders may have limited the statistical significance of this finding. Additionally, our logistic regression analysis revealed that being over the age of 55 significantly increased the risk of developing HZ, with an odds ratio of 2.55. This finding emphasizes the critical role of advanced age as a risk factor for HZ.

When examining the distribution of HZ between genders, it is reported that HZ is more common in women. This is often explained by the presence of comorbid conditions in women [26]. However, in a prospective study of 335,714 individuals, Opstelten et al. reported that female gender is an independent risk factor for HZ, regardless of other factors. Female gender has been specifically identified as a significant risk factor for HZ in individuals aged 25–64 [31]. In our study, it was found that women were significantly more frequently diagnosed with HZ than men, consistent with the literature. The female-to-male ratio was 1.25 in those with psychiatric diagnoses and 1.18 in those without. There was no significant difference between the two groups. The higher frequency of both psychiatric disorders and HZ in women may suggest that changes in the immune system, potentially acting through common pathways, could be involved in their etiology [31,32,33].

However, there are limitations to this study. Psychiatric disorder cases were identified based on diagnostic codes provided by physicians. Some potential psychiatric patients may have been misclassified as unaffected if they never sought help for their mental disorders, leading to potential misclassification that could skew results. HZ cases are generally diagnosed clinically by physicians. The silent clinical forms of HZ may have led to some cases being missed. Additionally, some non-HZ cases may have been incorrectly diagnosed as HZ, especially if not confirmed by laboratory diagnosis [34]. Certain antipsychotic medications may affect immune function, potentially impacting the observed association between psychiatric disorders and VZV reactivation [15]. Another limitation of this study is the potential impact of the COVID-19 pandemic on hospital admission rates during 2020. In Turkey, COVID-19 cases were widespread, and national health policies imposed strict lockdowns and restrictions to reduce the spread of the virus, particularly impacting non-emergency hospital admissions. This context likely contributed to a decrease in hospital visits, including for conditions like herpes zoster, during that period. Thus, the observed decrease in herpes zoster cases in 2020 may be more reflective of decreased healthcare access rather than a true reduction in incidence. This factor is acknowledged as a limitation in accurately interpreting the year-by-year trends observed in our findings [22,23,24].

Future research should focus on elucidating the mechanisms linking psychiatric disorders to immune dysregulation and increased susceptibility to herpes zoster (HZ). Studies that explore specific immune pathways—such as those involving Th17 and regulatory T cells—could provide insights into the role of immune changes in HZ reactivation among psychiatric patients. Additionally, examining the effects of psychotropic medications on immune function and HZ risk would help clarify potential contributing factors. Prospective cohort studies across various populations could further define causative relationships and inform the development of targeted interventions, including vaccination strategies and therapies to strengthen immune resilience in psychiatric populations.

## 5. Conclusions

Our findings suggest a positive relationship between psychiatric disorders and HZ. According to our results, physicians should be aware that HZ occurs more frequently in patients with psychiatric disorders. Evaluating other risk factors, such as age, in psychiatric patients and implementing preventive treatments could benefit public health. Given its efficacy in reducing the incidence and complications of HZ, vaccination should be considered a key component of preventive strategies. Future studies are needed to better identify the specific psychiatric disorders and the clinical presentation of HZ.

## Figures and Tables

**Table 1:** Yearly distribution of herpes zoster cases and gender differences (2019–2023).

Variables	2019	2020	2021	2022	2023	Total
**Number of hospital visits**	306,031	216,167	239,554	290,949	357,038	1,409,739
**Number of individuals diagnosed with HZ**	517	365	489	463	642	2476
**Number of women diagnosed with HZ**	305	201	264	224	368	1362
**Number of men diagnosed with HZ**	212	164	225	239	274	1114
**Female-to-male ratio in HZ cases**	1.4	1.2	1.1	0.9	1.3	1.2
**HZ annual prevalence among unique hospital visitors**	1.6 per 1000	1.6 per 1000	2 per 1000	1.5 per 1000	1.7 per 1000	
**Number of individuals who visited psychiatry department**	15,918	8646	8252	11,800	11,379	55,994
**Number of cases with both psychiatric disorder and HZ (%)**	49 (3 per 1000)	24 (2.7 per 1000)	55 (6.6 per 1000)	36 (3 per 1000)	61 (5.3 per 1000)	

HZ: herpes zoster.

**Table 2:** Comparison of age, gender, and psychiatric diagnosis frequency with HZ.

Variables	With Psychiatric Diagnosis	Without Psychiatric Diagnosis	χ^2^ (Deviation Value)	*p*-Value
Number of individuals diagnosed with HZ (%) *	225 (0.0040)	2251 (0.0016)	3678.08 ^a^	0.001
**Median age (IQR)**	50 (21.5)	55 (27)	−1.63 ^b^	0.11
**Female-to-male ratio**	1.25	1.18	158.20 ^a^	0.61

* HZ: herpes zoster, IQR: interquartile range, ^a^: Chi-square deviation value, ^b^: Mann–Whitney U.

**Table 3:** Anxiety, depressive, psychotic, and bipolar disorders in individuals diagnosed with both herpes zoster and psychiatric disorders.

Diagnosis	F-Statistic	Case (n)	Percentage with HZ Diagnosis	*p*-Value
Anxiety disorder	0.5329	153	5.78%	<0.001
Depressive disorder	0.5329	67	2.52%	<0.001
Psychotic disorder or Bipolar disorder	0.5329	5	0.19%	<0.001

HZ: herpes zoster.

**Table 4:** Logistic regression analysis.

Variables	B	Lower CI	Upper CI	Odds Ratio	*p*-Value
**Depressive disorder**	0.746	1.650	2.697	2.1	<0.001
**Psychotic disorder or bipolar disorder**	1.880	2.710	15.852	6.55	<0.001
**Anxiety disorder**	1.201	2.918	3.785	3.23	<0.001
**Gender/Female**	0.412	1.399	1.630	1.39	<0.001
**Age (>55)**	1.012	2.751	2.966	2.55	
**constant**	−5.924				

## Data Availability

All data obtained are available in an excel form that can be shared upon request.
